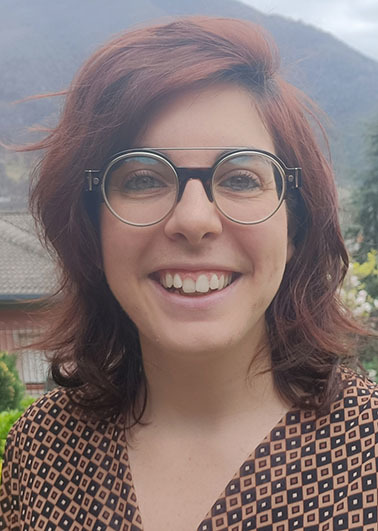# α_V_β_6_ Integrin: An Intriguing Target for COVID‐19 and Related Diseases

**DOI:** 10.1002/cbic.202100209

**Published:** 2021-06-15

**Authors:** Kelly Bugatti

**Affiliations:** ^1^ Dipartimento di Scienze degli Alimenti e del Farmaco Università di Parma Parco Area delle Scienze 27A 43124 Parma Italy

**Keywords:** α_V_β_6_, COVID-19, fibrosis, RGD, SARS-CoV-2

## Abstract

The outbreak of SARS‐CoV‐2 has been an extraordinary event that constituted a global health emergency. As the novel coronavirus is continuing to spread over the world, the need for therapeutic agents to control this pandemic is increasing. α_V_β_6_ Integrin may be an intriguing target not only for the inhibition of SARS‐CoV‐2 entry, but also for the diagnosis/treatment of COVID‐19 related fibrosis, an emerging type of fibrotic disease which will probably affect a significant part of the recovered patients. In this short article, the possible role of this integrin for fighting COVID‐19 is discussed on the basis of recently published evidence, showing how its underestimated involvement may be interesting for the development of novel pharmacological tools.

## Graphical abstract

**An emerging tool against the pandemic war**: Thanks to the identification of the RGD sequence on the spike protein of SARS‐CoV‐2, RGD‐recognizing integrins have recently received important attention. Among them, α_V_β_6_ may be an intriguing target not only for the inhibition of SARS‐CoV‐2 entry, but also for the diagnosis/treatment of COVID‐19 related fibrosis.

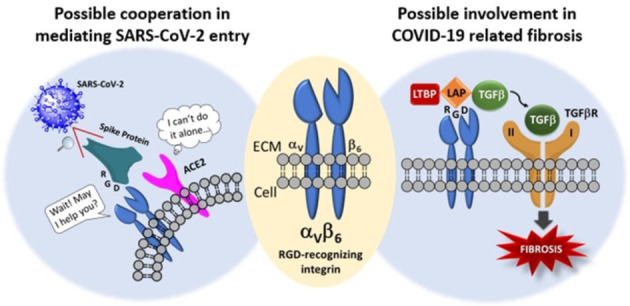

## 1 Introduction

SARS‐CoV‐2 is a highly contagious virus that has caused serious health crisis, resulting into a global pandemic situation. At the beginning of 2021, several agencies approved the first vaccines, and others will be hopefully soon commercialized.^[1]^ The extremely fast discovery and scale‐up production of these vaccines was really impressive but, on the other hand, no effective small‐molecules directly targeting SARS‐CoV‐2 have been commercialized yet, despite some compounds which can inhibit both the infection and replication of SARS‐CoV‐2 have been developed.^[2]^ In addition, discrete successes have been reached in treating the resulting problems caused by COVID‐19 using well‐known commercialized drugs (e. g. dexamethasone in hospitalized patients with COVID‐19).^[3]^ As consequence, continuing search for new pharmacological targets is extremely important to achieve marketable drugs at short notice, and therefore several drug targets and potential treatment have been suggested.^[4]^ In this context, α_V_β_6_ integrin may be an interesting target both for inhibiting SARS‐CoV‐2 entry and for the treatment of COVID‐19 related fibrosis. Integrin α_V_β_6_ is one of the RGD (Arg‐Gly‐Asp) recognizing integrins – heterodimeric cell surface receptors which mediate cellular communication – which is not expressed in healthy adult epithelia, but it is overexpressed in many epithelial aggressive tumours, as well as in pulmonary and liver fibrosis.[^5^, ^6^]

In the following paragraphs, the role of α_V_β_6_ in SARS‐CoV‐2 infection will be briefly described, starting from the supposed involvement in the virus internalization, going on to the role of this integrin in the development of COVID‐19 related lung fibrosis, and finally introducing promising compounds for the early detection of the pulmonary fibrotic state.

## 2 Targeting α_V_β_6_ for the Inhibition of SARS‐CoV‐2 Entry

ACE2 (angiotensin converting enzyme 2) was firstly identified as the primary receptor mediating SARS‐CoV‐2 cell entry by interacting with the spike protein of the virus,^[7]^ but recent evidence have shown how this internalization process may be more complex than first expected.^[8]^ Generally, the spike proteins of coronaviruses are known as some of the largest ones identified, which means that different domains within a single spike protein may interact with several receptors, leading to a very complicated mechanism of virus internalization.^[9]^ The involvement of RGD‐recognizing integrins (including α_V_ receptors)^[10]^ in facilitating the entry of several viruses in the host cell has been described, and it has therefore hypothesized for SARS‐CoV‐2.^[11]^ In point of fact, the RGD motif in the spike protein of SARS‐CoV‐2 has been identified as located outside and adjacent to its interaction interface with ACE2.^[12]^ Therefore, some authors propose that this domain may provide a complementary cell entry, despite the full mechanism is not fully clarified and only some hypothesis have been suggested, such as:^[13]^ *(i)* when the receptor binding domain (RBD) of the spike protein undergoes hinge‐like conformational shifts, the RGD motif is exposed to the surface of the host cell membrane and, once interacting with integrin, ACE2 may be recruited to the binding complex, facilitating the virus entry; *(ii)* the RGD motif of the spike protein may bind to the integrin parallelly (or sequentially) in an ACE2‐independent manner.^[12]^

In addition, it has been shown that SARS‐CoV‐2 reduces the ACE2 expression, which is an interesting contradiction, since ACE2 has been described as the main receptor mediating virus internalization.^[14]^ The cooperation with RGD‐binding integrins expressed on human airway epithelial cells (including α_V_β_3_, α_V_β_5_, α_V_β_6_ and α_V_β_8_) may explain this evidence: the RGD motif in the spike protein of SARS‐CoV‐2 may promote viral infection in low ACE2‐expressing cells,^[15]^ accordingly to the second hypothesized mechanism cited before.

Furthermore, the highly infectious variant B.1.1.7 of SARS‐CoV‐2 – recently reported in the UK – is characterized by multiple mutations, including the single‐amino‐acid replacements N501Y, which is located in the receptor‐binding domain. This substitution appears, on one hand, to increase affinity to ACE2 but, on the other hand, this replacement may cause a greater surface exposure of the RGD motif. In fact, N501 is located immediately distal to the RGD motif and the replacement of asparagine by tyrosine at that position could enhance the accessibility of the RGD motif for integrin‐binding.^[8]^ This mutation supports the hypothesis that integrins may be involved in SARS‐CoV‐2 entry, although it should be taken into account that future mutations could, on the contrary, reduce the accessibility and the exposure of the RGD domain.

Although α_V_β_6_ integrin is expressed only in case of damaged epithelia, some authors have suggested that the presence of chronic and/or inflammation condition, which leads to integrin overexpression, may relate to an increase of virus infectivity.^[16]^ Additionally, it has been hypothesized that, after the early stages of the infection, the viral replication fast accelerates, compromising the epithelial‐endothelial barriers. This damage may promote the integrin expression, including α_V_β_6_, increasing virus infectivity.^[8]^

**Figure 1:**
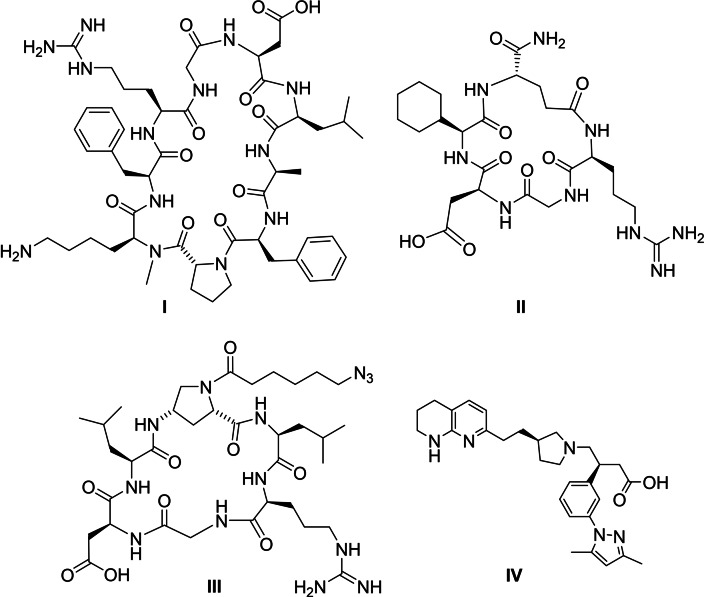
Structure of the nonapeptide antagonist *c*[FRGDLAFp(NMe)K] (**I**, α_V_β_6_ IC_50_ 0.26 nM, solid phase binding assay), the cyclopeptide *c*(RGD‐Chg‐E)‐CONH_2_ (**II**, α_V_β_6_ IC_50_ 1.6 nM, competitive ELISA assay), the peptidomimetic *c*(Amp)LRGDL (**III**, α_V_β_6_ IC_50_ 8.3 nM, solid phase receptor binding assay) and of the non‐peptidic small‐molecule compound developed by GSK (**IV**, α_V_β_6_ pIC_50_ 8.4, GSK cell adhesion assays). Compound **III** possesses a terminal azide group, which can be exploited as anchoring point for the development of a covalent conjugates, as mentioned above.

Based on this evidence, the administration of compounds blocking α_V_β_6_ binding as free ligands or, better, in combination with ACE2 inhibitors, may provide a promising avenue to impair the entry of virus. Interestingly, George and colleagues suggested that novel antifibrotic drugs targeting α_V_β_6_ may prevent the development of severe SARS‐CoV‐2 infection.^[17]^ Potent and selective α_V_β_6_ integrin ligands have been recently published in literature,^[18]^ such as the nonapeptide α_V_β_6_ antagonist *c*[FRGDLAFp(NMe)K] (I, Figure 1),^[19]^ the cyclopeptide *c*(RGD‐Chg‐E)‐CONH_2_ (II, Figure 1),^[20]^ the peptidomimetic *c*(Amp)LRGDL (III, Figure 1)^[21]^ and the non‐peptidic small‐molecule inhibitor developed by GSK (IV, GSK3008348, Figure 1, discussed in the following paragraph).^[22]^

Considering that no small‐molecule drugs for SARS‐CoV‐2 infection have been approved till now, it might be interesting to study more in‐depth these integrin ligands, to open new perspectives in solving this global emergency. Indeed, if no small‐molecule targeting ACE2 have been clinically developed yet, – considering how fast this field of research is running – it might be pondered to use complementary/alternative ways to operate. Perhaps, the simultaneous targeting of multiple ACE2 domains may be an intriguing solution. Novel infection blockers can be designed to be highly compatible with the spike protein to block either integrin binding, ACE2 binding or the putative cooperation between them. Concerning this last point, *ad‐hoc* dual covalent conjugates^[23]^ may be rationally designed, with the aim to simultaneous target both α_V_β_6_ integrin and ACE2.

However, the regulation of integrin receptors by means of a synthetic ligand could be very challenging, – as demonstrated by the historically failure of the cyclopeptide cilengitide^[24]^ – due to several reasons, for instance *(i)* the possibility of these receptors to assume different conformational states, which are related to different biological activities and may impact on the agonist/antagonist behaviour of the ligand; *(ii)* more interestingly, the “excess” of the ligand‐selectivity toward a specific integrin subtype may cause the activation of an alternative compensatory pathway mediated by other integrins.^[25]^ In spite of that, the increasing number of integrin‐targeted ligand in different clinical trials^[26]^ and the presence of integrin‐targeted drugs on the market give hope to this intriguing field of research.

## 3 Targeting α_V_β_6_ for the Treatment of COVID‐19 Related Fibrosis

Despite many people surviving COVID‐19, it is possible that the virus left traces of its transit even in recovered patients: it has been hypothesized that a third of the survivors who have been infected with SARS‐CoV‐2 will develop significant pulmonary fibrosis.^[27]^ This means that in a not‐too‐distant future, we could have a high number of fibrotic patients, and we have to be prepared for the treatment of this emerging disease.

According with the suggestion by George et al.^[17]^ it could be appropriate to evaluate the use of compounds developed for the Idiopathic Pulmonary Fibrosis (IPF) in treating COVID‐19 related fibrosis. At the moment, only two drugs are approved for the treatment of IPF, namely pirfenidone and nintedanib, but they unfortunately only slow the disease progression. In fact, they attenuate the rate of lung function decline by about 50 % with different mechanism of action, while improving life expectancy only by 2–5 years. Considering that the request of antifibrotic drugs will probably increase in the near future, investing in the development of new drugs for the treatment of fibrotic related disease is needed as never before.

Pulmonary fibrosis (including the COVID‐19 relate) has been described as a consequence of a cytokine storm. In particular, the pathologic evolution of the Adult Respiratory Destress Syndrome (ARDS) – developed approximately by the 5–8 % COVID‐19 patients – is thought to involve three overlapping phases: exudative, proliferative, and fibrotic. The exudative phase is characterized by the release of proinflammatory cytokines and the endothelial/epithelial barrier disruption, and it is followed by the exudative and fibroproliferative phases. Here, fibrocytes, fibroblasts, and myofibroblasts accumulate in the alveolar compartment, leading to excessive deposition of matrix components and to the consequently development of pulmonary fibrosis.^[28]^

Among all the involved cytokines, Transforming Growth Factor β (TGFβ) is a central mediator of fibrogenesis, since it is upregulated, and it mediates fibroblast phenotype and function. Additionally, its activation is correlated to worsen prognosis in several fibrotic diseases, including pulmonary fibrosis.^[29]^ As a consequence, TGFβ is an ideal pharmacological target, although the direct inhibition of such pleiotropic and multifunctional cytokine may lead to possible severe side effects, and it is therefore preferable to target TGFβ activation or signalling pathway.^[30]^

**Figure 2:**
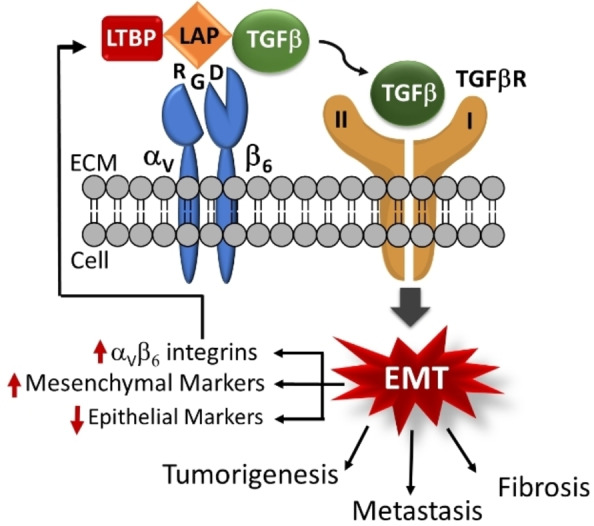
Schematic representation of α_V_β_6_‐mediated activation of TGFβ. α_V_β_6_ Integrin recognizes the RGD sequence in the Latency‐Associated Peptide (LAP) of the TGFβ latent complex; the interaction RGD‐receptor causes conformational modifications which finally produce the release of active TGFβ into the extracellular environment, and the consequently binding to its receptor. ECM, Extracellular Matrix; LTBP, Latency TGFβ Binding Protein; LAP, Latency Associated Peptide; RGD, Arg‐Gly‐Asp sequence; EMT, Epithelial‐To‐Mesenchymal Transition.

Interestingly, TGFβ is activated by α_V_ integrins, in particular α_V_β_6_.^[31]^ When this cytokine binds the TGFβ receptor, the stimulation of the intracellular signals leads to the so‐called Epithelial‐To‐Mesenchymal Transition (EMT), ultimately promoting tumorigenesis, metastasis, and fibrosis. Additionally, TGFβ activation leads to the promotion of the α_V_β_6_ expression, causing a sort of vicious circle which sustains and aggravates the EMT (Figure 2). For these reasons, α_V_β_6_ has emerged as an interesting target for the treatment of pulmonary fibrosis. Moreover, recent data suggest that high levels of integrin α_V_β_6_ correlates with high mortality of fibrotic patients.^[6]^

Accordingly, the evaluation of emerging drugs targeting α_V_β_6_ as therapeutic agents could hold promise in treating the COVID‐19 related fibrosis,^[17]^ and some advanced compounds may be interesting candidates. For instance, the already mentioned non‐peptide small‐molecule GSK3008348 (IV, Figure 1) has successfully reached the phase II clinical trial for the treatment of Idiopathic Pulmonary Fibrosis.^[32]^ This compound has been demonstrated to reduce downstream pro‐fibrotic TGFβ signals to normal levels in human IPF lungs, to induce rapid internalization and lysosomal degradation of the α_V_β_6_ integrin in human lung epithelial cells, to promote prolonged inhibition of TGFβ signalling and to reduce lung collagen deposition in murine bleomycin‐induced lung fibrosis model. These promising data suggest that GSK3008348 may be beneficial also for the treatment of COVID‐19 related disease, although this hypothesis has yet to be proven.

In addition, another promising dual small molecule targeting α_V_β_6_/α_V_β_1_ integrins – developed for the treatment of IPF and fibrotic diseases and which showed to reduce lung TGFβ activity in healthy volunteers – is currently in Phase II studies for the treatment of patients with ARDS associated with at least severe COVID‐19, under the name of NCT04565249.^[33]^ Moreover, interesting compounds have been published also in the field of macromolecules: the humanized α_V_β_6_ monoclonal antibody STX100 gave promising results for the treatment of IPF, and it has recently completed phase II trial (under the name of BG00011).^[34]^ This macromolecule demonstrated TGFβ suppression by reduction of pSMAD2, one of the main signal transducers for TGFβ.

Other small molecules or antibodies targeting α_V_β_6_ **–** and, in general, α_V_ integrins – have been proposed as potential therapeutic agents against different types of fibrosis:^[35]^ in this perspective, using this interesting “armory” to fight new types of fibrosis are worth exploring.

## 4 Targeting α_V_β_6_ for the Early Diagnosis of COVID‐19 Related Fibrosis

It is reasonable to expect that patients severely affected by COVID‐19 will develop pulmonary fibrosis, but what about the other survivors, who maybe had mild/no symptoms? Will they develop fibrosis? The answer to this question is uncertain, so that the monitoring of the pulmonary state of those patients and the early diagnosis of fibrotic conditions are primary epidemiological objectives.

In this context, non‐invasive molecularly targeted imaging tools may play a key role for the early diagnosis of fibrotic lesions. As mentioned before, α_V_β_6_ is expressed at low levels or undetectable in healthy adult epithelium, but it is soon upregulated in injured tissues, including fibrotic lung; this characteristic makes this receptor an ideal target for non‐invasive imaging techniques. Recently, a fluorinated analogue of the α_V_β_6_‐binding peptide A20FMDV2 ([^18^F]‐FB‐A20FMDV2) has been studied as PET tracer in both healthy and fibrotic lungs^[36]^ and the study concluded that lung uptake of [^18^F]‐FB‐A20FMDV2 was markedly increased in subjects with pulmonary fibrosis in comparison with healthy volunteers. Based on this evidence, Foster et al.^[37]^ obtained the first human PET/CT images in a patient after 2 months of the acute phase of SARS‐CoV‐2 infection, using a [^18^F]α_V_β_6_‐BP,^[38]^ the 4‐[^18^F]fluorobenzyl ([^18^F]FBA)‐labelled peptide that is currently under phase I clinical study (under the name NCT03164486) in patients with different type of cancers. This study showed a correlation between α_V_β_6_‐targeted [^18^F]‐α_V_β_6_‐BP PET and lung damage, which was identified by CT; however, the main limitations of this study are the reduced number of patients and the single imaging time point. The authors have already scheduled [^18^F]‐α_V_β_6_‐BP PET/CT scans for 3 and 6 months, and 10 patients will be enrolled in the study. This follow‐up will be critically important to evaluate the persistence and potential progression of abnormalities in lungs and other organs.

Moreover, another integrin α_V_β_6_‐recognizing cystine knot tracer, the [^18^F]FP‐R01‐MG‐F2 (clinically called NCT03183570), which has been recently developed for the detection of cancer and IPF,^[39]^ is undergoing phase I clinical study^[40]^ for the PET/CT detection of α_V_β_6_ in IPF, in Primary Sclerosing Cholangitis and, in particular, in COVID‐19 related fibrosis.

In conclusion, the possibility to early diagnose the fibrotic state in the lung of the recovered COVID‐19 patients is extremely important to prevent the severe onset of the disease, and this will be made possible by the availability of precise and safe diagnostic tools. In this context, the efforts to develop α_V_β_6_ targeting compounds for the early fibrosis state detection are intensifying and some promising compounds are currently at an advanced stage of development. Since the number of patients requiring this early monitoring will soon increase, there is an ongoing need to investigate and to evaluate new diagnostic tools is mandatory.

## 5 Conclusions

SARS‐CoV‐2 continues to infect millions of people worldwide, and the road to the discovery of an effective cure for COVID‐19 is still long. All the potential biological targets involved in the entry and spread of the virus should be carefully evaluated. Among them, α_V_β_6_ integrin appears to be of interest both for the inhibition of SARS‐CoV‐2 entry and for the treatment of COVID‐19 related disease. Investing time and research on this fascinating target may help in solving the global pandemic problem from multiple angles.

## Conflict of interest

The authors declare no conflict of interest.

## Biographical Information

*Kelly Bugatti is currently a postdoctoral researcher in the bioorganic chemistry research group of Prof. Dr. Franca Zanardi, at the University of Parma, Italy (Food and Drug Department). She graduated in Pharmaceutical Chemistry and Technology at the University of Parma in 2017, and she obtained her Ph.D. in Drugs, Biomolecules and Health Products at the same university (2021) under the supervision of Prof. Dr. Lucia Battistini. Her research interests are focused on the design and synthesis of integrin‐targeted small molecule peptidomimetics and their covalent conjugates for biomedical applications*.